# Intraoperative blood loss as a predictor of outcomes in liver transplantation: determining optimal cutoff values for improved graft survival

**DOI:** 10.1007/s00423-025-03898-z

**Published:** 2025-11-05

**Authors:** Ayato Obana, Miho Akabane, Khalid Mumtaz, Kejal Shah, Matthew Hamilton, Rithin Punjala, Austin Schenk, Navdeep Singh, Sylvester Black, Kenneth Washburn, Musab Alebrahim

**Affiliations:** 1 Comprehensive Transplant Center, Department of Surgery, Ohio State University Wexner Medical Center, Ohio , OH USA; 2 Comprehensive Transplant Center, Department of Hepatology, Ohio State University Wexner Medical Center, Ohio, OH USA; 3 Department of Hepato-Biliary-Pancreatic and Gastrointestinal Surgery, School of Medicine, International University of Health and Welfare, Chiba, Japan

**Keywords:** Cutoff, Estimated blood loss, Graft survival, Liver transplantation, Risk factor

## Abstract

**Background:**

Liver transplantation (LT) remains the definitive treatment for end-stage liver disease, with intraoperative estimated blood loss (EBL) receiving limited attention despite its potential impact on outcomes. This study investigated the impact of EBL on graft survival (GS) in LT recipients and aimed to identify a clinically optimal EBL cutoff to guide surgical management.

**Methods:**

This observational cohort study analyzed 914 adult patients who underwent primary orthotopic LT at Ohio State University Wexner Medical Center between January 2016 and December 2023. Intraoperative EBL was calculated by subtracting the volume of salvaged blood from the total volume lost during surgery, then normalized by dividing by the patient’s body weight, resulting in adjusted EBL (aEBL). The primary outcome was GS, defined as the time from transplantation to graft failure, re-LT, or death. Kaplan-Meier analysis and Cox regression were used to evaluate GS, and a restricted cubic spline with five knots was applied to determine the optimal aEBL cutoff.

**Results:**

Multivariate analysis confirmed aEBL as an independent risk factor for 1-year GS (HR:1.01, 95%CI:1.00-1.01, *p* < 0.001) and 3-year GS (HR:1.01, 95%CI:1.00-1.01, *p* < 0.001). The optimal aEBL cutoff was established at 25.0 mL/kg. Patients with aEBL < 25.0 mL/kg demonstrated superior GS rates at 90 days (*p* = 0.03), 1 year (*p* = 0.007), and 3 years (*p* = 0.003) compared to those with aEBL ≥ 25.0 mL/kg. Higher MELD-Na scores (OR:1.07, 95%CI:1.05–1.09, *p* < 0.001) and DCD donor status (OR:1.61, 95%CI:1.13–2.29, *p* = 0.01) were significant predictors of exceeding this threshold.

**Conclusions:**

This study establishes aEBL as an independent risk factor for GS in LT recipients and identifies 25.0 mL/kg as a significant cutoff impacting both short-term and long-term outcomes. These findings underscore the importance of tailoring blood loss management to individual patient characteristics, particularly body weight, and suggest a practical approach to enhance outcomes for LT recipients.

**Supplementary Information:**

The online version contains supplementary material available at 10.1007/s00423-025-03898-z.

## Introduction

Liver transplantation (LT) remains the definitive treatment for end-stage liver disease, with over 9,000 procedures performed annually in the United States [1]. The demand for LT continues to rise, driven in part by advancements in donor organ preservation techniques, such as normothermic regional perfusion and normothermic machine perfusion, which have significantly increased donor liver utilization rates [2]. Despite ongoing donor shortages, much of the current focus has been on optimizing donor-recipient matching and post-operative immunosuppression strategies [3–6]. However, intraoperative factors, particularly estimated blood loss (EBL), have received comparatively limited attention. Notably, the United Network for Organ Sharing (UNOS) database lacks comprehensive data on intraoperative EBL, and this important factor has been underrepresented in the scientific literature.

Prior research has shown that intraoperative blood transfusion is associated with increased morbidity and mortality in living donor LT recipients [7]. LT recipients often present with thrombocytopenia and coagulation factor deficiencies, leading to the frequent need for massive transfusion during surgery [8, 9]. In some cases, more than 150 units of blood products were required [10]. Risk prediction models, such as the McCluskey index, have been developed to identify patients at high risk for massive transfusion during liver transplantation [11]. Intraoperative transfusion management relies on close collaboration with anesthesiologists, employing cell salvage techniques and thromboelastography to guide transfusion decisions [12, 13]. Massive transfusion is reportedly associated with significant complications, including coagulopathy, transfusion-related acute lung injury, acute kidney injury, and increased infection risk [14]. These complications are known to contribute to early post-transplant mortality and poorer outcomes, potentially impairing graft function and reducing survival [15, 16]. Donor characteristics, particularly donation after circulatory death (DCD) status, may also influence intraoperative bleeding patterns. DCD donors, who experience warm ischemia following cardiac arrest before organ procurement, represent an increasingly important organ source to address donor shortages but may present unique surgical challenges compared to donation after brain death (DBD) donors due to ischemia-reperfusion injury and potential tissue compromise.

To date, while some studies have examined the impact of massive transfusion in relation to intraoperative EBL, limited data exist on the specific impact of EBL itself. Moreover, clinicians have traditionally relied on their own experience regarding the optimal amount of intraoperative EBL during LT, as no globally established guideline for the optimal EBL amount exists. We hypothesized that a more precise understanding of intraoperative EBL could help ensure more favorable graft survival (GS). Therefore, this study aims to investigate the impact of EBL on GS in primary LT and identify the clinically optimal EBL cutoff to guide surgical management.

## Methods

### Study design

This observational cohort study was conducted to evaluate the impact of intraoperative EBL on GS in LT recipients. The study was performed at the Ohio State University Wexner Medical Center (OSUMC) and included data spanning from January 2016 to December 2023.

### Study population

The cohort comprised adult patients (aged ≥ 18) who underwent initial orthotopic LT during the study period. Patients who received multiple organ transplants, re-LT, or living donor LT were excluded from the analysis.

### Data source

Data for this study were obtained from the electronic medical records (EMR) maintained by OSUMC. The dataset included detailed information on patient demographics, clinical characteristics, transplant details, and follow-up outcomes. Key variables collected included recipient age, sex, body mass index (BMI), Model for End-Stage Liver Disease including Sodium (MELD-Na), race, operative time, days in the Intensive Care Unit (ICU) post-LT, length of total hospital stay post-LT, past surgical history of upper abdomen, use of Transjugular Intrahepatic Portosystemic Shunt (TIPS), etiology of end stage liver disease (ESLD), donor age, BMI, cold ischemic time (CIT), hepatitis C virus (HCV) antibody status, DCD, and the use of OrganOx for machine perfusion, as OSUMC exclusively uses OrganOx for normothermic machine perfusion when machine perfusion is applied. Additionally, intraoperative transfusion data including red blood cells (RBC), fresh frozen plasma (FFP), platelets, and cryoprecipitate units were collected for correlation analysis with EBL.

### Definition of aEBL

Intraoperative EBL was calculated by subtracting the volume of salvaged, purified, and returned blood via cell salvage techniques from the total volume of blood lost during the surgical procedure. This net value was defined as the intraoperative EBL. Additionally, as part of a sub-analysis, we examined total blood loss, including the blood salvaged by the cell saver, to provide a more comprehensive assessment of intraoperative bleeding. The primary variable of interest, intraoperative EBL, was normalized by dividing it by the patient’s body weight, resulting in a derived metric termed “adjusted EBL (aEBL)” [17]. All liver transplants at our institution routinely employed intraoperative cell salvage techniques with standardized protocols for blood recovery and reinfusion. Transfusion management followed institutional blood management practices with real-time surgical–anesthesia team coordination and laboratory-based triggers. These standardized protocols may vary across transplant programs.

### Outcomes

The primary outcome of interest was GS, defined as the time from transplantation to graft failure, re-LT, or death. All analyses were conducted with approval from the institutional review board at OSUMC (No. 2023H0392). All research was conducted in accordance with both the Declarations of Helsinki and Istanbul.

### Statistical analyses

All statistical analyses were conducted using Python 3.12.4 (https://www.python.org/). Donor and recipient demographics were reported as frequencies with percentages or as median values with interquartile range (IQR). Differences between categorical values were estimated using the chi-square test, while differences between continuous values were assessed with the Mann-Whitney U or Kruskal-Wallis tests as appropriate. Additionally, independent t-tests were employed to compare the means of continuous variables between two groups.

The Kaplan-Meier method was employed to evaluate GS, and the Log-rank test was used to compare the survival curves. Cox regression analysis was used for multivariate analysis to calculate the hazard ratio (HR) for graft loss. To better understand the relationship between aEBL and HR for graft loss, a restricted cubic spline curve with five knots was applied. Both non-adjusted and adjusted HR curves were drawn using the same set of variables from the Cox regression model. The optimal aEBL cutoff point was determined by identifying the highest absolute Z-statistic [17]. A two-tailed p-value of less than 0.05 was considered statistically significant. We also performed nonparametric bootstrapping (B = 500). In each bootstrap sample, an adjusted Cox model with restricted cubic splines for aEBL was fitted, and the threshold along a 0–100 mL/kg grid at which the spline Wald |Z| first exceeded 1.96 was recorded. We summarized the bootstrap-selected thresholds by median, IQR, and 2.5th–97.5th percentiles. Analyses were repeated for both the primary aEBL definition (subtracting salvaged blood) and total blood loss (including salvaged blood). Spearman correlation coefficients were calculated to assess the relationship between aEBL and intraoperative transfusion requirements.

## Results

### Patient characteristics

**Fig. 1:**
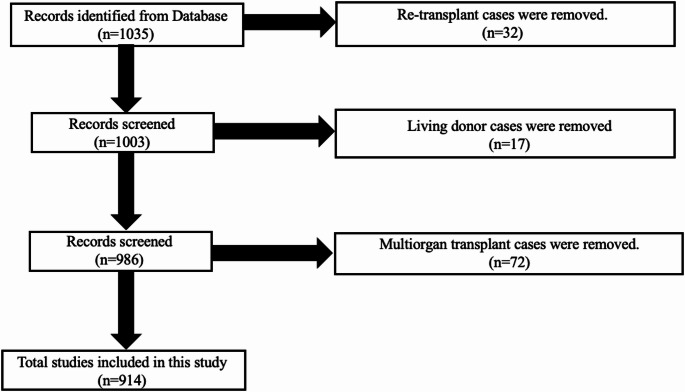
Flow diagram of patient selection for the study. Of 1,035 patients identified from the database, 914 patients who underwent primary orthotopic liver transplantation were included after excluding re-transplant (*n* = 32), living donor (*n* = 17), and multiorgan transplant cases (*n* = 72)

The initial cohort comprised 1,035 patients. After applying exclusion criteria (living donor LT, re-LT, and multi-organ transplant recipients), the final study population consisted of 914 patients undergoing primary orthotopic LT (Fig. 1).

The study cohort (*n* = 914) had a median follow-up period of 33 months (IQR: 14–53 months). The demographic characteristics were as follows: the median age was 57 years (IQR: 48–64), with a male predominance (*n* = 564, 61.7%), and the majority were Caucasian (*n* = 821, 89.8%) (Table 1). The median BMI was 29.0 kg/m² (IQR: 25.0–33.7). Transplant-specific metrics included a median MELD-Na score of 23 (IQR: 18–29). Alcohol-related liver disease (*n* = 328, 35.6%) and nonalcoholic fatty liver disease (NAFLD) (*n* = 250, 27.3%) were the two most common etiologies of end-stage liver disease. The median operative time was 5.02 h (IQR: 4.20–5.95).

**Table 1:** Characteristics of patients enrolled in this study

	aEBL < 25.0 (*N* = 247)	aEBL ≥ 25.0 (*N* = 667)	Total (*N* = 914)	*p*-value
Recipient				
Age, y (IQR)	58 (50–63)	57 (48–63)	57 (48–64)	0.37
Male, n (%)	156 (63.2)	408 (61.2)	564 (61.7)	0.64
BMI, kg/m^2^ (IQR)	31.0 (26.7–35.3)	28.6 (24.9–32.9)	29.0 (25.0–33.7)	< 0.001
MELD-Na at LT (IQR)	15 (10–23)	25 (20–30)	23 (18–29)	< 0.001
Race, n, (%)				0.62
White	225 (91.1)	596 (89.4)	821 (89.8)	
Black	11 (4.5)	41 (6.1)	52 (5.7)	
Hispanic	5 (2.0)	13 (1.9)	18 (2.0)	
Asian	5 (2.0)	14 (2.1)	19 (2.1)	
Others	1 (0.1)	3 (0.3)	4 (0.4)	
Operative time, hour (IQR)	4.42 (3.87–5.12)	5.30 (4.44–6.17)	5.02(4.20–5.95)	< 0.001
Estimated blood loss, L (IQR)	1.5 (1.0–2.0)	5.0 (3.0–8.0)	4.0 (2.0–7.0)	< 0.001
Days in ICU after LT, day (IQR)	1.5 (1.0–2.5)	2.0 (1.2–3.0)	1.7 (1.1–2.7)	< 0.001
Length of total hospital stay after LT, day, (IQR)	8.0 (6.0–11.0)	10.0 (8.0–14.0)	9.0 (7.0–13.0)	0.001
Blood recovered by cell saver, L, (IQR)	0.7 (0.3–1.0)	1.3 (0.7–2.4)	1.1 (0.5–2.2)	< 0.001
PSH of upper abdomen, n, (%)	14 (5.7)	34 (5.1)	48 (5.3)	0.86
TIPS, n, (%)	19 (7.7)	53 (7.9)	72 (7.9)	1.00
Etiology, n, (%)				0.77
Alcohol	57 (36.5)	271 (35.7)	328 (35.6)	
NAFLD	46 (29.5)	204 (26.9)	250 (27.3)	
Viral	19 (12.2)	88 (11.6)	107 (11.7)	
Autoimmune	18 (11.5)	83 (10.9)	101 (11.0)	
Others	14 (9.0)	94 (12.4)	108 (11.8)	
Malignancy	2 (1.3)	19 (2.5)	21 (2.3)	
**Donor**				
Age, y (IQR)	40 (29–54)	40 (30–53)	40 (30–53)	0.74
BMI, kg/m^2^ (IQR)	28 (24–32)	27.4 (23.8–32.6)	27.4 (23.8–32.6)	0.12
CIT, minutes, (IQR)	294 (234–378)	294 (240–396)	294 (240–387)	0.37
HCV antibody positive, n, (%)	27 (10.9)	92 (13.8)	119 (13.0)	0.30
DCD donor, n, (%)	65 (26.3)	209 (31.3)	274 (30.0)	0.17
Use of OrganOx, n, (%)	13 (5.3)	52 (7.8)	65 (7.1)	0.24

NOTE: Continuous variables: median [IQR]; Categorical variable: number (%)

*Abbreviations:* *EBL *Estimated blood loss, *BMI* Body mass index, *CIT* Cold ischemic time, *IQR* Interquartile range, *MELD-Na* Model for End-Stage Liver Disease including Sodium, *Ab* Antibody, *LT* Liver transplantation, *DCD* Donation after circulatory death, *NAFLD* Nonalcoholic Fatty Liver Disease, *ICU* Intensive care unit, *TIPS* Transjugular intrahepatic portosystemic shunt, *PSH* Past surgical history, *HCV* Hepatitis C virus

Post-transplantation care metrics showed a median ICU stay of 1.7 days (IQR: 1.1–2.7) and a median length of total hospital stay of 9.0 days (IQR: 7.0–13.0). Donor characteristics were as follows: median age of 40 years (IQR: 30–53), median BMI of 27.4 kg/m² (IQR: 23.8–32.6), and median CIT of 294 min (IQR: 240–387). Notably, 274 (30.0%) cases involved DCD donors, while the OrganOx perfusion system was utilized in 65 (7.1%) cases.

### Impact of aEBL on GS

Univariate Cox regression analysis showed that aEBL was significantly associated with both 1-year GS (HR:1.01, 95%CI [confidence interval]: 1.00–1.01, *p* < 0.001) and 3-year GS (HR:1.01, 95%CI: 1.00–1.01, *p* < 0.001) (Table 2). Multivariate Cox regression analysis further demonstrated that advanced recipient age (HR:1.07, 95%CI: 1.04–1.10, *p* < 0.001), aEBL (HR:1.01, 95%CI: 1.00–1.01, *p* < 0.001), length of stay at ICU (HR:1.04, 95%CI: 1.01–1.06, *p* = 0.001), and etiology of cirrhosis categorized as “others” (HR:2.48, 95%CI: 1.12–5.43, *p* = 0.02) were independent risk factors for 1-year GS. For 3-year GS, recipient age (HR:1.06, 95%CI: 1.03–1.08, *p* < 0.001), aEBL (HR:1.01, 95%CI: 1.00–1.01, *p* < 0.001), operative time (HR:1.21, 95%CI: 1.05–1.40, *p* = 0.01), length of stay at ICU (HR:1.03, 95%CI: 1.03–1.05, *p* = 0.01), and etiology of cirrhosis categorized as “others” (HR:2.26, 95%CI: 1.14–4.27, *p* = 0.01) were also significant. Both univariate and multivariate analyses confirmed that aEBL is a significant risk factor for 1- and 3-year GS.

**Table 2:** Univariate and multivariate analysis for GS at 1 year and 3 years

GSat 1 year	Univariate			Multivariate		
Variable	HR	95% CI	*p*-value	HR	95% CI	*p*-value
Recipient						
Male	0.76	0.46–1.26	0.28	0.61	0.46–1.06	0.08
Age	1.05	1.02–1.08	< 0.001	1.07	1.04–1.10	< 0.001
BMI	1.00	0.96–1.04	0.96	1.01	0.97–1.06	0.57
MELD-Na at LT	1.01	0.98–1.04	0.60	1.02	0.98–1.05	0.31
aEBL	1.01	1.00–1.01	< 0.001	1.01	1.00–1.01	< 0.001
Operative time	1.44	1.27–1.64	< 0.001	1.20	0.99–1.43	0.051
Days in ICU after LT	1.05	1.03–1.07	< 0.001	1.04	1.01–1.06	0.001
PSH of upper abdomen	0.92	0.29–2.93	0.88	1.34	0.89–4.38	0.63
TIPS	0.39	0.10–1.60	0.19	0.28	0.17–1.19	0.08
Etiology						
Autoimmune	1.05	0.48–2.31	0.90	1.69	0.41–4.28	0.27
Malignancy	0.69	0.10–4.98	0.71	0.21	0.21–2.13	0.18
NAFLD	1.11	0.64–1.93	0.70	0.98	0.70–2.01	0.95
Viral	0.85	0.37–1.98	0.71	1.16	0.39–3.03	0.76
Others	1.64	0.86–3.16	0.14	2.48	1.12–5.43	0.02
**Donor**						
DCD	1.17	0.68–1.99	0.58	1.01	0.50–2.00	0.76
HCV antibody positive	0.71	0.31–1.66	0.44	0.76	0.60–1.82	0.54
CIT	1.00	0.99–1.00	0.16	1.01	0.99–1.00	0.36
Donor Age	1.00	0.99–1.02	0.65	0.99	0.97–1.01	0.54
Use of OrganOx	1.35	0.54–3.36	0.53	0.94	0.54–3.84	0.94
**GS at 3 years**	**Univariate**			**Multivariate**		
**Variable**	**HR**	**95% CI**	**p-value**	**HR**	**95% CI**	**p-value**
**Recipient**						
Male	0.95	0.62–1.44	0.80	0.78	0.64–1.23	0.29
Age	1.05	1.02–1.07	< 0.001	1.06	1.03–1.08	< 0.001
BMI	1.00	0.97–1.03	1.00	1.00	0.97–1.04	0.97
MELD-Na at LT	1.00	0.98–1.02	0.97	1.01	0.98–1.04	0.41
aEBL	1.01	1.00–1.01	< 0.001	1.01	1.00–1.01	< 0.001
Operative time	1.36	1.22–1.53	< 0.001	1.21	1.05–1.40	0.03
Days in ICU after LT	1.05	1.03–1.07	< 0.001	1.03	1.03–1.05	0.01
PSH of upper abdomen	0.80	0.29–2.18	0.66	0.92	0.29–2.55	0.87
TIPS	0.53	0.20–1.45	0.22	0.41	0.10–1.12	0.08
Etiology						
Autoimmune	0.66	0.42–1.03	0.29	0.96	0.31–2.25	0.92
Malignancy	1.27	0.40–4.01	0.69	0.87	0.10–3.20	0.84
NAFLD	1.09	0.70–1.70	0.71	0.95	0.64–1.69	0.85
Viral	1.11	0.61–2.04	0.73	1.34	0.42–2.73	0.42
Others	1.58	0.92–2.71	0.10	2.26	1.14–4.27	0.01
**Donor**						
DCD	1.28	0.83–1.96	0.27	1.22	0.68–1.97	0.76
HCV antibody positive	1.01	0.44–2.35	0.96	1.11	0.57–2.04	0.54
CIT	1.00	0.99–1.00	0.24	1.00	0.99–1.00	0.42
Donor Age	1.01	0.99–1.02	0.65	1.00	0.99–1.02	0.90
Use of OrganOx	1.27	0.51–3.17	0.60	0.90	0.54–3.32	0.87

NOTE: Continuous variables: median [IQR]; Categorical variable: number (%)

*Abbreviations:* a*EBL* Adjusted estimated blood loss, *BMI* Body mass index, *CIT* Cold ischemic time, *MELD-Na* Model for End-Stage Liver Disease including Sodium, *DCD* Donor from circulatory death, *GS* Graft Survival, *NAFLD* Nonalcoholic Fatty Liver Disease, *TIPS* Transjugular intrahepatic portosystemic shunt, *PSH* Past surgical history, *HCV*: Hepatitis C virus, *CI*: Confidence Interval.

### Optimal cutoff of aEBL for predicting GS

**Fig. 2:**
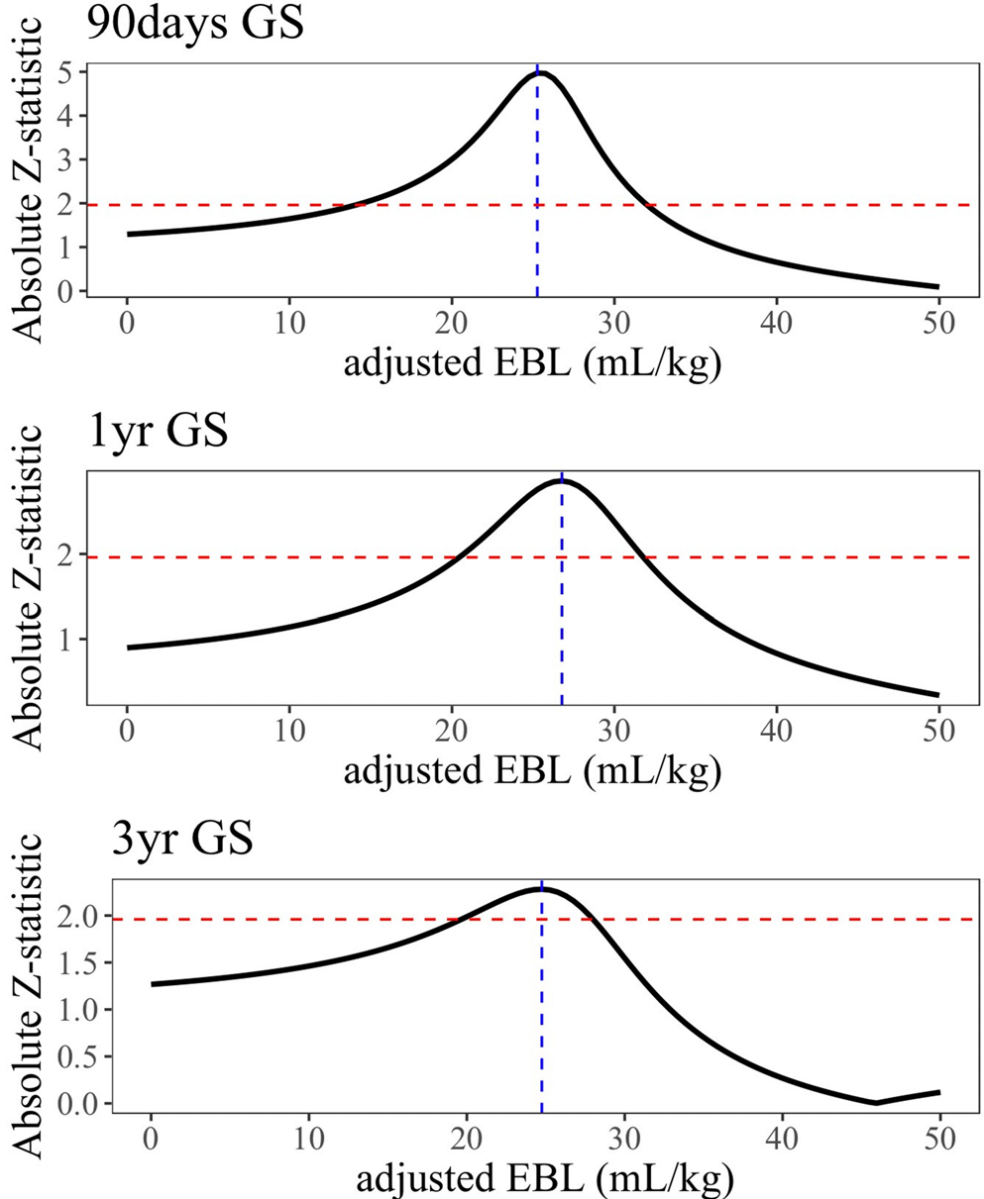
Restricted cubic spline curves showing the relationship between aEBL and GS. (**a**) Unadjusted hazard ratios and (**b**) Adjusted hazard ratios for graft loss at 90 days, 1 year, and 3 years after LT. Gray shading represents 95%CIs

**Fig. 3:**
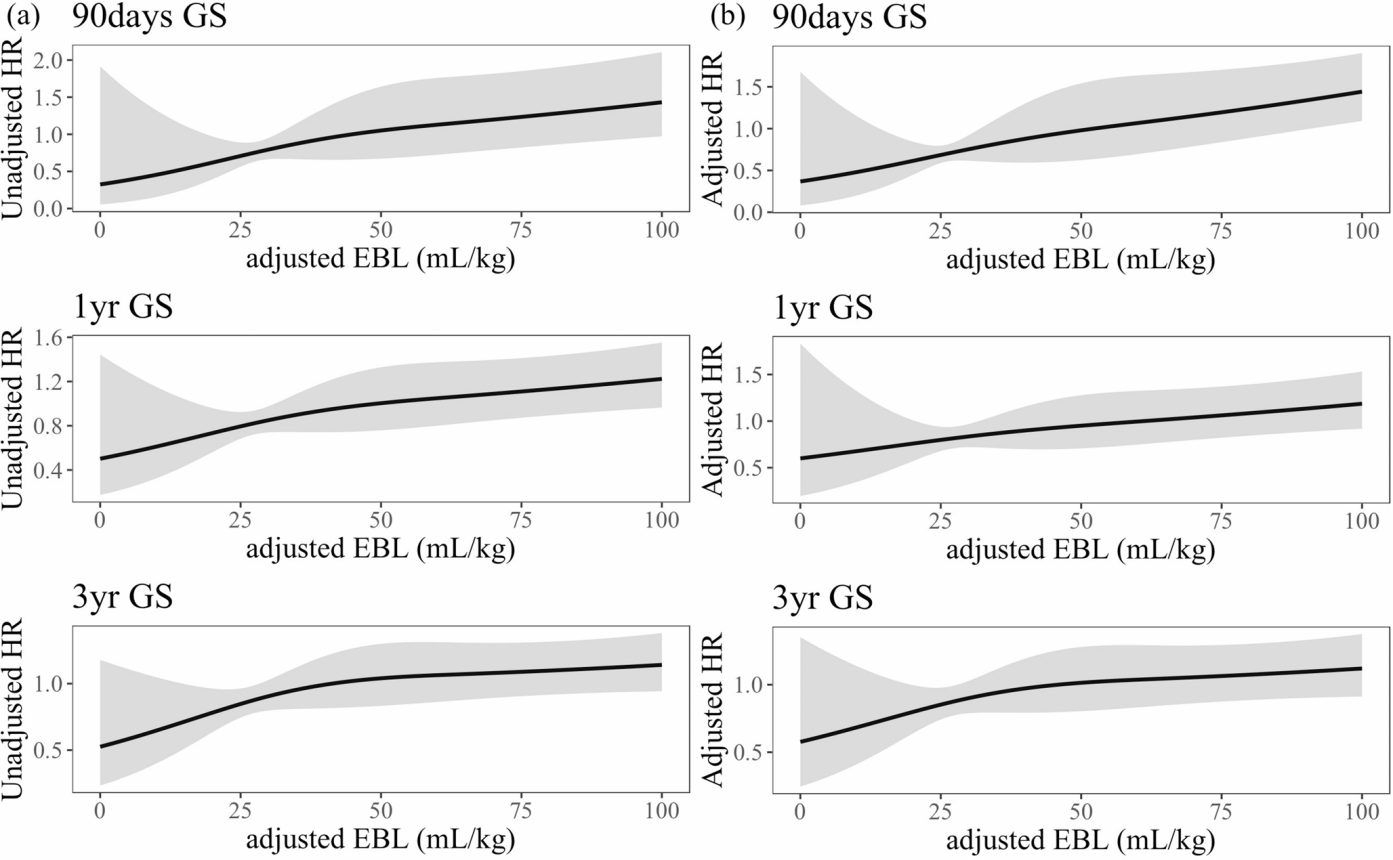
Absolute Z-statistics for identifying optimal aEBL cutoff values at 90 days, 1 year, and 3 years after LT. The blue dotted vertical lines indicate the maximum absolute Z-statistic values, and the red dotted horizontal lines represent the reference values. Maximum Z-statistic values were observed at 25.3, 26.8, and 24.7 mL/kg for 90 days, 1 year, and 3 years, respectively

The unadjusted restricted cubic spline curve demonstrated a linear increase in HR corresponding to rising aEBL. This linear relationship persisted even after adjusting for independent risk factors identified in the multivariable Cox regression analysis (Fig. 2a and b). To identify the optimal cutoff value for aEBL, we analyzed trends in absolute Z-statistics at 90 days, 1 year, and 3 years post-transplantation (Fig. 3). The maximum absolute Z-statistic values were observed at 25.3, 26.8, and 24.7 mL/kg of aEBL for 90 days, 1 year, and 3 years, respectively, resulting in the establishment of a cutoff value of 25.0 mL/kg. This threshold was subsequently assessed for its predictive accuracy in stratifying the risk of graft loss.

### GS based on aEBL threshold

**Fig. 4:**
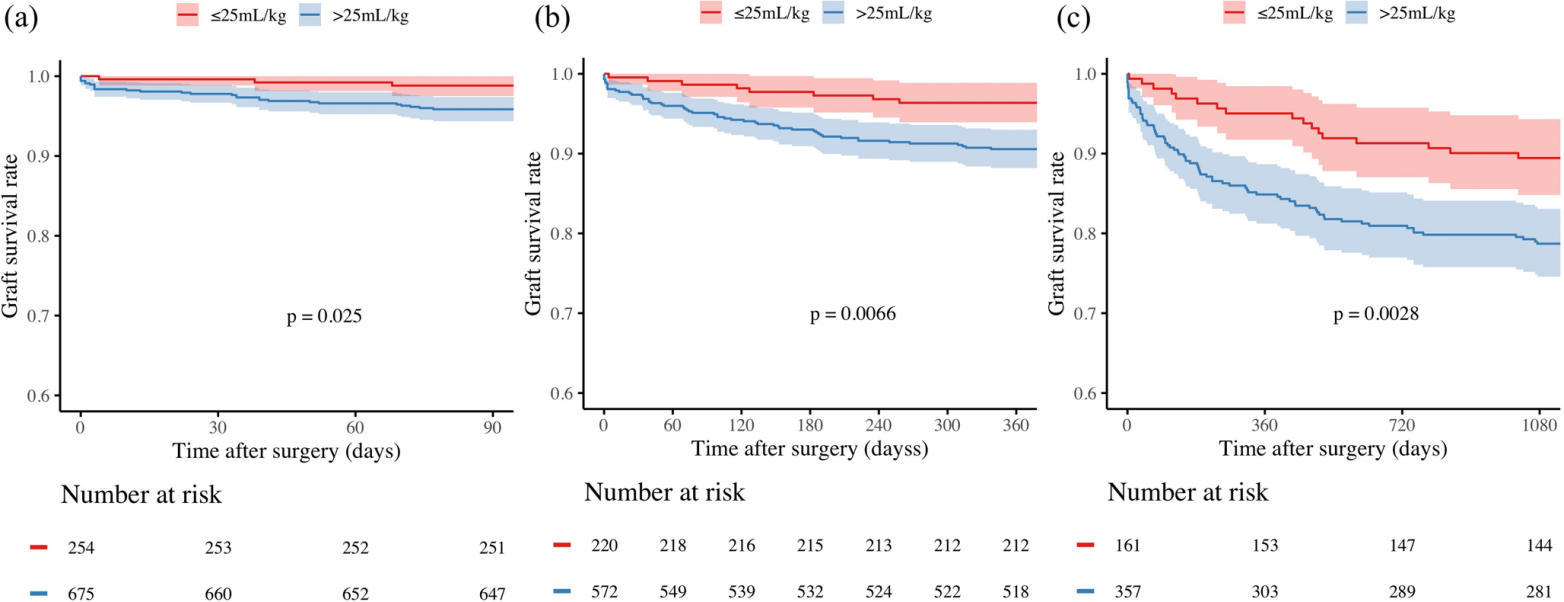
Kaplan-Meier survival curves comparing GS between patients with aEBL < 25mL/kg and ≥ 25mL/kg. (**a**) 90-day GS, (**b**) 1-year GS, and (**c**) 3-year GS. Shaded areas represent 95% CI. The number of patients at risk is shown below each graph

Kaplan-Meier survival analyses demonstrated significant differences in GS between patients with aEBL < 25.0 mL/kg and those with aEBL ≥ 25.0 mL/kg (Fig. 4). These differences were evident at 90 days (*p* = 0.03), 1 year (*p* = 0.007), and 3 years (*p* = 0.003). The cohort with aEBL < 25.0 mL/kg consistently showed superior GS rates compared to the cohort with aEBL ≥ 25.0 mL/kg. Bootstrap validation of the primary analysis (aEBL excluding salvaged blood) revealed a median threshold of 21 mL/kg (IQR 12–25, 95% bootstrap percentile interval 0–32). Our pragmatic cutoff of 25 mL/kg lies near the upper quartile of this distribution, supporting its clinical applicability.

To further characterize the cohort with aEBL < 25.0 mL/kg, patients were stratified into two groups: the low aEBL group (aEBL < 25.0 mL/kg, *n* = 247) and the high aEBL group (aEBL ≥ 25.0 mL/kg, *n* = 667) (Table 1). Significant differences were observed between the groups in several parameters. The patients in the low aEBL group had a significantly higher BMI (31.0 [IQR: 26.7–35.3] vs. 28.6 [IQR: 24.9–32.9] kg/m^2^, *p* < 0.001), while those in the high aEBL group had significantly higher MELD-Na scores (25 [IQR: 20–30] vs. 15 [IQR: 10–23], *p* < 0.001). Operative time was also longer in the high aEBL group (5.30 [IQR: 4.44–6.17] vs. 4.42 [IQR: 3.87–5.12] hours, *p* < 0.001). Postoperative outcomes also differed, with the high aEBL group experiencing longer ICU stays (2.0 [IQR: 1.2–3.0] vs. 1.5 [IQR: 1.0–2.5] days, *p* < 0.001) and extended total hospital stays (10.0 [IQR: 8.0–14.0] vs. 8.0 [IQR: 6.0–11.0] days, *p* = 0.001).

To identify independent risk factors associated with exceeding the established aEBL threshold of 25.0 mL/kg, a multivariate logistic regression analysis was performed (Table 3). The analysis revealed that a higher MELD-Na score (OR: 1.07, 95%CI: 1.05–1.09, *p* < 0.001) and DCD donor status (OR: 1.61, 95%CI: 1.13–2.29, *p* = 0.01) were significant predictors of surpassing this threshold.

**Table 3:** Multivariate logistic regression analysis to identify risk factors associated with surpassing the aEBL cutoff of 25.0 mL/kg

	Odds ratio	95% Cl	*p*-value
Recipient			
Age	1.00	0.98–1.01	0.69
Male	0.92	0.67–1.27	0.63
MELD-Na at LT	1.07	1.05–1.09	< 0.001
Race			
Black	Reference		
White	0.51	0.26–1.02	0.06
Hispanic	0.39	0.11–1.40	0.15
Others	0.66	0.21–2.11	0.48
PSH of upper abdomen	0.84	0.43–1.63	0.61
TIPS	0.90	0.51–1.60	0.72
**Donor**			
Age	1.00	0.99–1.02	0.37
BMI, kg/m^2^	1.00	0.98–1.02	0.99
CIT	1.00	0.99–1.00	0.35
HCV antibody positive	1.43	0.88–2.30	0.14
DCD donor	1.61	1.13–2.29	0.01
Use of OrganOx	2.08	0.88–4.93	0.09

NOTE: Continuous variables: median [IQR]; Categorical variable: number (%)

*Abbreviations:* a*EBL* Adjusted estimated blood loss, *BMI* Body mass index, *CIT* Cold ischemic time, *MELD-Na* Model for End-Stage Liver Disease including Sodium, *DCD* Donor from circulatory death, *HCV* Hepatitis C virus, *PSH* Past surgical history, *TIPS* Transjugular intrahepatic portosystemic shunt, *CI *Confidential interval

### Transfusion requirements and correlation with aEBL

Analysis of intraoperative transfusion requirements demonstrated significant differences between aEBL groups (Supplementary Table 1). Patients with aEBL ≥ 25.0 mL/kg had higher transfusion requirements for RBC (median 7 [IQR: 5–10] vs. 6 [IQR: 3–7] units, *p* < 0.001), FFP (6 [IQR: 4–10] vs. 2 [IQR: 2–6] units, *p* < 0.001), platelets (2 [IQR: 2–4] vs. 2 [IQR: 2–2] units, *p* < 0.001), and cryoprecipitate (4 [IQR: 2–5] vs. 4 [IQR: 2–4] units, *p* = 0.02) compared to those with aEBL < 25.0 mL/kg. Strong positive correlations were observed between aEBL and RBC transfusion requirements (Spearman ρ = 0.26, *p* < 0.001), with an even stronger correlation when RBC units were normalized by body weight (ρ = 0.33, *p* < 0.001). In an exploratory multivariable model that additionally included intraoperative RBC units, greater RBC volume was associated with surpassing the cutoff (OR 1.11 per unit, *p* < 0.001; Supplementary Table 2); because transfusion is downstream of blood loss, this model was not used for primary causal inference.

### Sub-analysis considering total EBL including salvaged blood

We conducted a subgroup analysis that incorporated total EBL, including the blood salvaged by the cell saver. In this analysis, total blood loss per body weight (aEBL including salvaged blood) had a median of 66.5 mL/kg [IQR: 37.0–113.4.]. Both the unadjusted and adjusted spline curves (Supplementary Figs. 1a and 1b) for 90-day, 1-year, and 3-year GS showed a similar increase in HR as aEBL (including salvaged blood) increased. The maximum absolute Z-statistic occurred at aEBL of 37.4, 35.9, and 33.8 mL/kg for 90-day, 1-year, and 3-year GS, respectively (Supplementary Fig. 2). By setting a threshold of 35.0 mL/kg for risk stratification, we observed significant differences in GS at 90 days, 1 year, and 3 years, indicating effective risk stratification (Supplementary Fig. 3). Bootstrap validation of total blood loss (including salvaged blood) demonstrated a median threshold of 30 mL/kg (IQR 0–38, 95% bootstrap percentile interval 0–73), showing greater variability compared to the primary analysis.

## Discussion

This study investigated the impact of intraoperative EBL on GS in LT recipients and identified an optimal cutoff value for clinical use. While previous studies have focused on predicting massive transfusion using risk indices such as the McCluskey score [11], our findings demonstrated that aEBL, which normalizes intraoperative EBL by body weight, was an independent risk factor for GS in LT recipients. Moreover, our results suggest that an aEBL of 25.0 mL/kg could serve as an optimal benchmark to ensure favorable GS following LT. This cut-off value holds significant potential as a clinically useful indicator for intraoperative blood management during LT.

Efforts to maximize liver graft utilization have traditionally focused on preoperative donor-recipient matching [3–5] and post-operative management, such as immunosuppression regimens [6], postoperative care [18] and nutritional support [19]. While previous research has highlighted the influence of intraoperative blood transfusion on postoperative GS [20], the direct impact of blood loss itself has not been extensively discussed in LT. This gap in the literature may be partially due to the absence of intraoperative data, such as blood loss volume and operative time, in the UNOS database, both of which could negatively affect GS. Although the impact of EBL on GS has been rarely reported in orthotopic LT, several studies have demonstrated its negative effects in living donor LT [7] and hepatectomy [17]. However, these studies did not account for body weight, which significantly affects blood loss outcomes [21]. To address this limitation, we used the aEBL metric, providing a more precise assessment of blood loss relative to patient characteristics. In hepatobiliary surgery for perihilar cholangiocarcinoma, aEBL has been shown to significantly affect postoperative outcomes, with a proposed optimal cutoff value [17]. Our study applied this concept to LT, investigating the impact of aEBL on GS and determining an optimal cutoff for clinical practice. Encouraging clinicians to monitor and optimize intraoperative blood loss could improve outcomes for LT recipients and potentially enhance graft utilization.

Our analysis confirmed that aEBL was an independent risk factor for GS. Notably, patients in the low aEBL group had a significantly higher BMI, which likely reflects that heavier patients have a lower aEBL when blood loss is normalized by body weight. Conversely, patients in the high aEBL group had significantly elevated MELD-Na scores, possibly due to difficulties in controlling intraoperative bleeding in patients with advanced liver failure, who have reduced coagulation factors and platelets [22]. This presumably led to prolonged surgical duration, necessitating massive transfusions due to substantial blood loss, which subsequently increased the risk of postoperative complications [14], and extended ICU and total hospital stays [23]. Our finding that aEBL, adjusted for body size, remained a significant risk factor for GS suggests that a more tailored approach to intraoperative blood loss based on patient weight could have important clinical implications. Analysis of intraoperative transfusion requirements showed that patients with aEBL ≥ 25.0 mL/kg required substantially more blood products across categories, with strong positive correlations between aEBL and RBC units. In an exploratory multivariable model that additionally included intraoperative RBC units, greater RBC volume was associated with surpassing the cutoff (OR 1.11 per unit, *p* < 0.001; Supplementary Table 2). Because transfusion occurs downstream of blood loss, this exploratory model was used to contextualize resource needs and was not considered for primary causal inference; our primary model excluded transfusion variables. Taken together, these findings suggest that the weight-adjusted cutoff provides a practical benchmark for intraoperative management and blood bank planning while continuing to stratify graft-survival risk.

The current study demonstrated that aEBL threshold of 25.0 mL/kg significantly impacted GS post-LT. To illustrate, for an 80.0 kg recipient, this threshold translates to a blood loss cut-off of 2,000 mL. In contrast, the median EBL in our cohort was 4,000 mL, which is substantially higher than the 2,000 mL preferred based on the 25.0 mL/kg cutoff. A recent study from Nagoya University, focusing on perihilar cholangiocarcinoma, proposed a considerably lower cutoff of 10.0 mL/kg [17], which is markedly lower than our 25.0 mL/kg threshold. However, our higher threshold appears justifiable given the increased bleeding tendency in patients with end-stage liver disease [8, 9]. Our analysis indicated that both recipient disease severity, as measured by MELD-Na score, and donor characteristics, particularly DCD status, increase the likelihood of higher intraoperative blood loss during LT. This finding is consistent with previous reports demonstrating increased blood product requirements in DCD liver transplantation [24]. DCD liver grafts differ fundamentally from DBD grafts in that they undergo a period of warm ischemia following cardiac arrest, which can result in endothelial dysfunction, increased tissue friability, and compromised microvascular integrity [25]. These pathophysiological changes may necessitate more cautious perioperative management and meticulous surgical technique, potentially contributing to prolonged operative duration and increased bleeding risk throughout the transplant procedure. The 61% increased odds (OR: 1.61, 95%CI: 1.13–2.29) of exceeding the 25.0 mL/kg aEBL threshold in recipients of DCD grafts has important clinical implications. First, surgical teams should anticipate higher blood loss when utilizing DCD organs and ensure adequate preparation, including optimized blood bank resources and enhanced coagulation management strategies. Second, enhanced hemostatic techniques may be warranted during the hepatectomy phase when DCD grafts are used, including optimized use of energy devices and meticulous surgical technique to minimize blood loss. Third, anesthesiologists should be prepared for more intensive perioperative blood and fluid management in DCD transplant cases. Furthermore, given that DCD grafts are already associated with higher rates of biliary complications [26] and primary non-function [27] compared to DBD grafts, the additional burden of increased blood loss and its associated morbidity underscores the complexity of DCD liver transplantation. However, the continued utilization of DCD grafts remains essential to address the ongoing organ shortage, making optimization of surgical techniques and perioperative management strategies crucial to mitigate these additional risks while maintaining acceptable outcomes.

Clinically, when preoperative estimates or intraoperative measurements approach 2,000 mL of blood loss, it is crucial to collaborate with anesthesiologists and nursing staff to control further bleeding. This may involve managing central venous pressure [28, 29] and adjusting fluid balance [30]. Operative time demonstrated significance in univariate analysis for 1-year GS but showed only borderline significance in the multivariate model (HR: 1.20, 95%CI: 0.99–1.43, *p* = 0.051), suggesting that its effect may be partially mediated through its strong correlation with aEBL. Since hepatectomy typically accounts for the majority of blood loss in LT procedures [31], this finding emphasizes the independent predictive value of blood loss when controlling for other perioperative factors. In cases where substantial blood loss is anticipated, prioritizing hemostasis, and where safe, expediting the hepatectomy process may mitigate the additive adverse effects of both massive blood loss and prolonged operative time. Although bootstrap validation supported a central tendency between approximately 20–30 mL/kg, the dispersion—particularly for aEBL including salvaged blood (95% bootstrap percentile interval: 0–73 mL/kg)—suggests potential cohort specificity and the need for external validation. Thus, while this cutoff value was derived from a single-center cohort at OSUMC, the 25 mL/kg threshold should be considered hypothesis-generating pending multicenter validation studies.

Several limitations should be acknowledged. First, this single-center, retrospective study was conducted at a high-volume transplant center with standardized protocols that may not reflect practices at other institutions. Variations in blood loss measurement methods, transfusion management protocols, perioperative care pathways, and surgical techniques across different centers may limit the generalizability of our findings and the universal applicability of the 25.0 mL/kg cutoff value. Second, the retrospective nature of the study raises concerns about incomplete or inaccurate data recording, unmeasured confounding and potential selection bias. Third, our cohort included patients with various etiologies including those with acute liver failure who may require routine massive transfusion, potentially influencing our results. Fourth, this study specifically focused on the impact of estimated blood loss volume itself, rather than transfusion requirements or blood product administration patterns. While transfusion data may provide additional insights, our objective was to establish the direct relationship between actual blood loss and GS outcomes. Additionally, the observed survival differences may reflect early post-transplant events rather than sustained long-term effects of blood loss, as patients with massive bleeding often experience early complications that influence the entire follow-up period. Fifth, while we identified DCD status as a significant predictor of increased blood loss, we did not analyze specific technical factors that may contribute to this association, such as warm ischemia time, extent of vascular reconstruction required, or machine perfusion utilization patterns. In our cohort, OrganOx machine perfusion was more frequently utilized in DCD cases (15.3%[42/274] vs. 3.6%[23/640] in DBD cases, *p* < 0.001), which may influence bleeding patterns through altered graft characteristics. Future studies should explore these mechanistic factors, including the impact of machine perfusion technology, to better understand and potentially mitigate the increased bleeding risk associated with DCD liver transplantation. Sixth, while we normalized blood loss by body weight based on the physiological principle that circulating blood volume is approximately 65–75 mL/kg [21], the observation that patients in the low aEBL group maintained significantly higher BMI values despite normalization raises questions about the adequacy of simple weight-based adjustment. Patients with higher BMI may have proportionally different cardiovascular reserve and tolerance to blood loss compared to those with lower BMI [32]. Alternative normalization approaches, such as body surface area calculations or estimated blood volume formulas that account for body composition, might provide more precise physiological adjustment in future studies.

In conclusion, the current study identified aEBL as an independent risk factor for GS in LT recipients. We established a threshold of 25.0 mL/kg as a significant cutoff point, impacting both short-term and long-term GS outcomes. This finding underscores the importance of tailoring blood loss management to individual patient characteristics, particularly body weight. By focusing on aEBL as a modifiable risk factor, we suggest a practical approach to enhance both short-term and long-term outcomes for LT recipients. Future studies should aim to validate these findings and explore interventions to optimize blood loss management in LT, including phase-specific analysis of bleeding during different surgical stages.

## Supplementary Information

Below is the link to the electronic supplementary material.

Supplementary figure 1 (PNG 479 KB)

High Resolution Image (TIF 444 KB)

Supplementary figure 2 (PNG 749 KB)

High Resolution Image (TIF 984 KB)

Supplementary figure 3 (PNG 737 KB)

High Resolution Image (TIF 1.28 MB)

Supplementary figure 4 (PNG 248 KB)

High Resolution Image (TIF 374 KB)

Supplementary file 5 (20.7 KB)

## Data Availability

The datasets generated and analyzed during the current study are available from the corresponding author upon reasonable request.
