# Supplementary figures and images for: Intraoperative blood loss as a predictor of outcomes in liver transplantation: determining optimal cutoff values for improved graft survival
Source: Langenbecks Arch Surg. 2025 Nov 5;411(1):3. doi: 10.1007/s00423-025-03898-z (PMC12589207; doi:10.1007/s00423-025-03898-z)

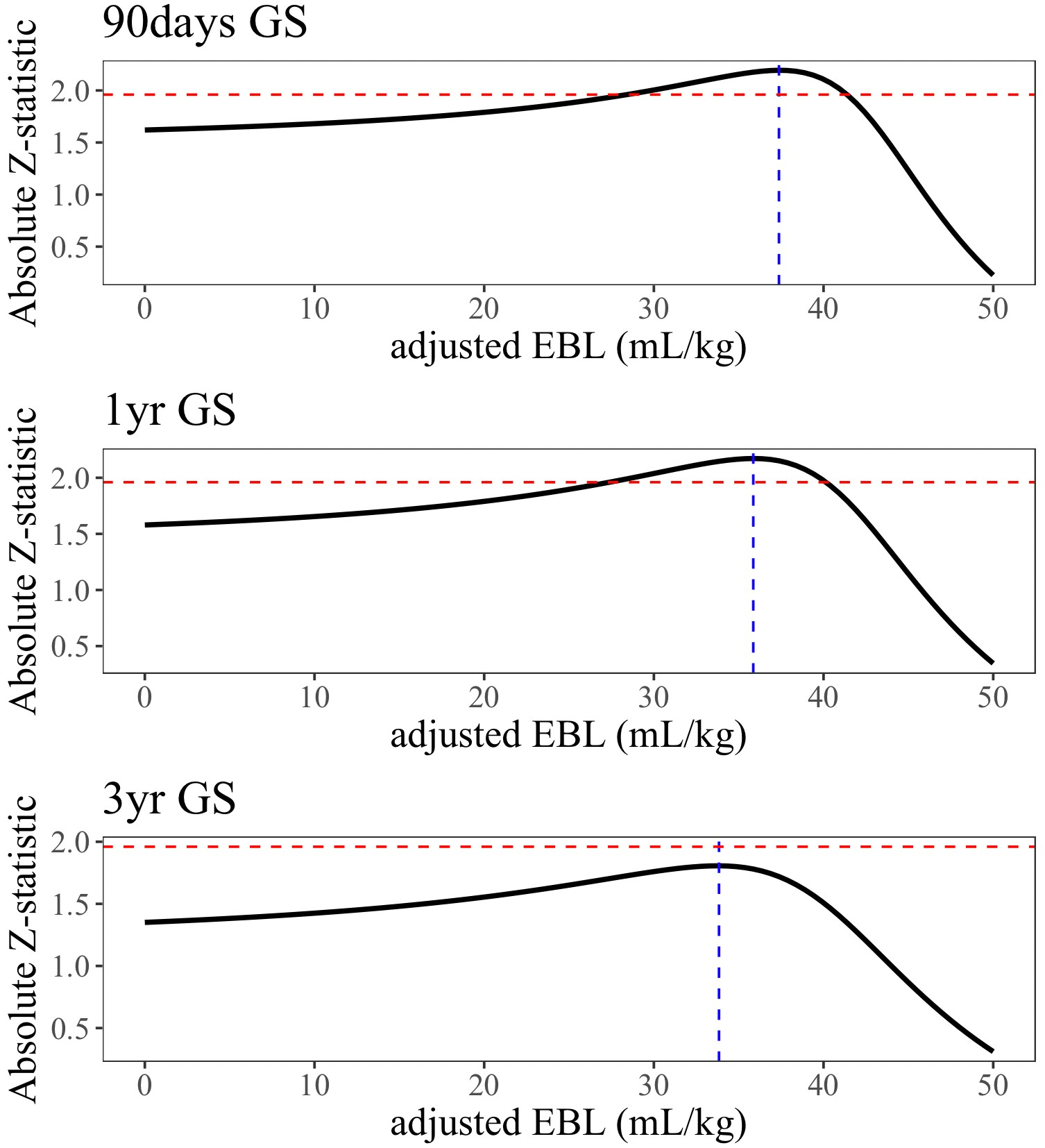

Supplement: Supplementary file 1 — (PNG 479 KB) [file 423_2025_3898_Fig5_ESM.png]

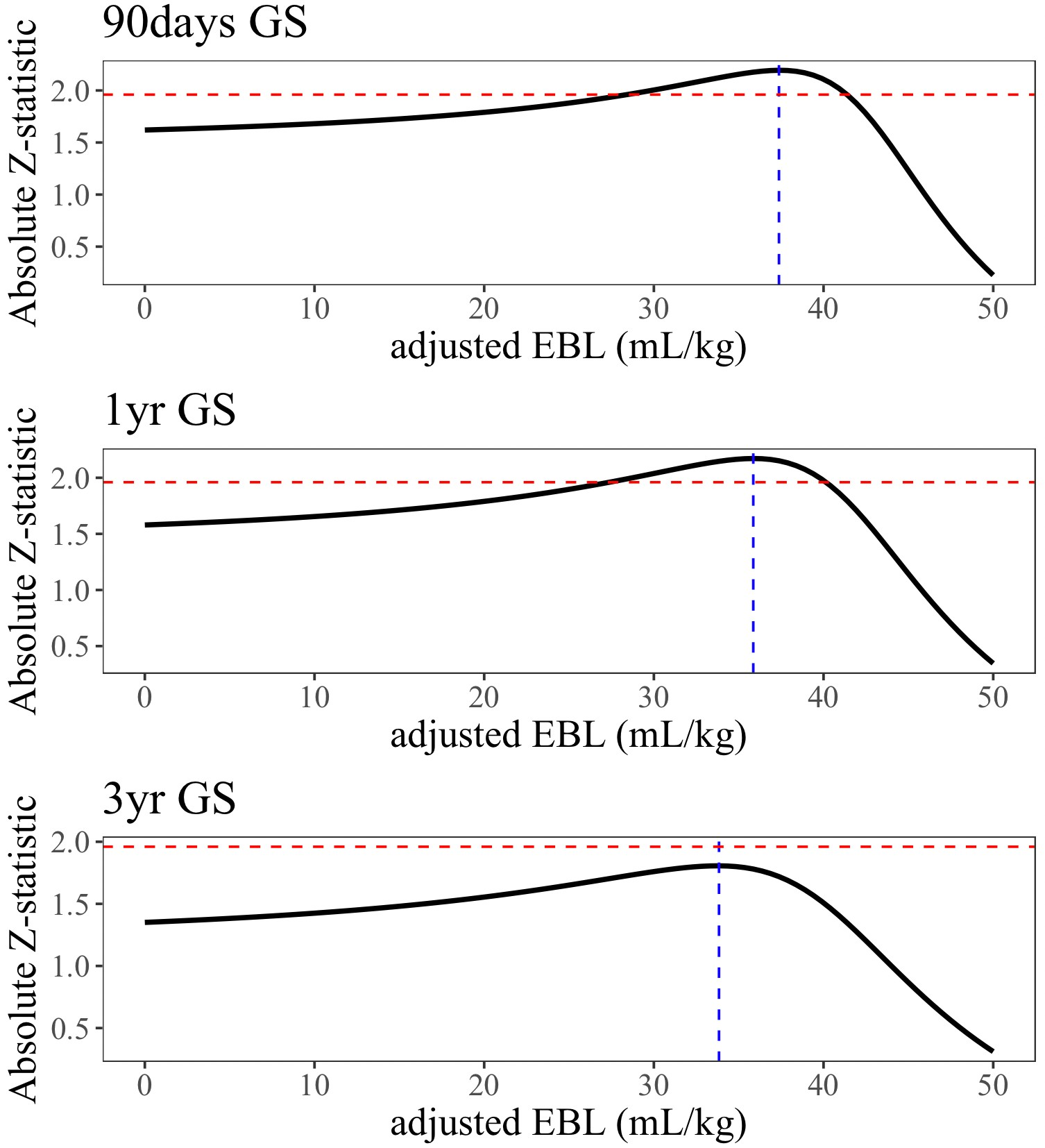

Supplement: Supplementary file 2 — High Resolution Image (TIF 444 KB) [file 423_2025_3898_MOESM1_ESM.tiff]

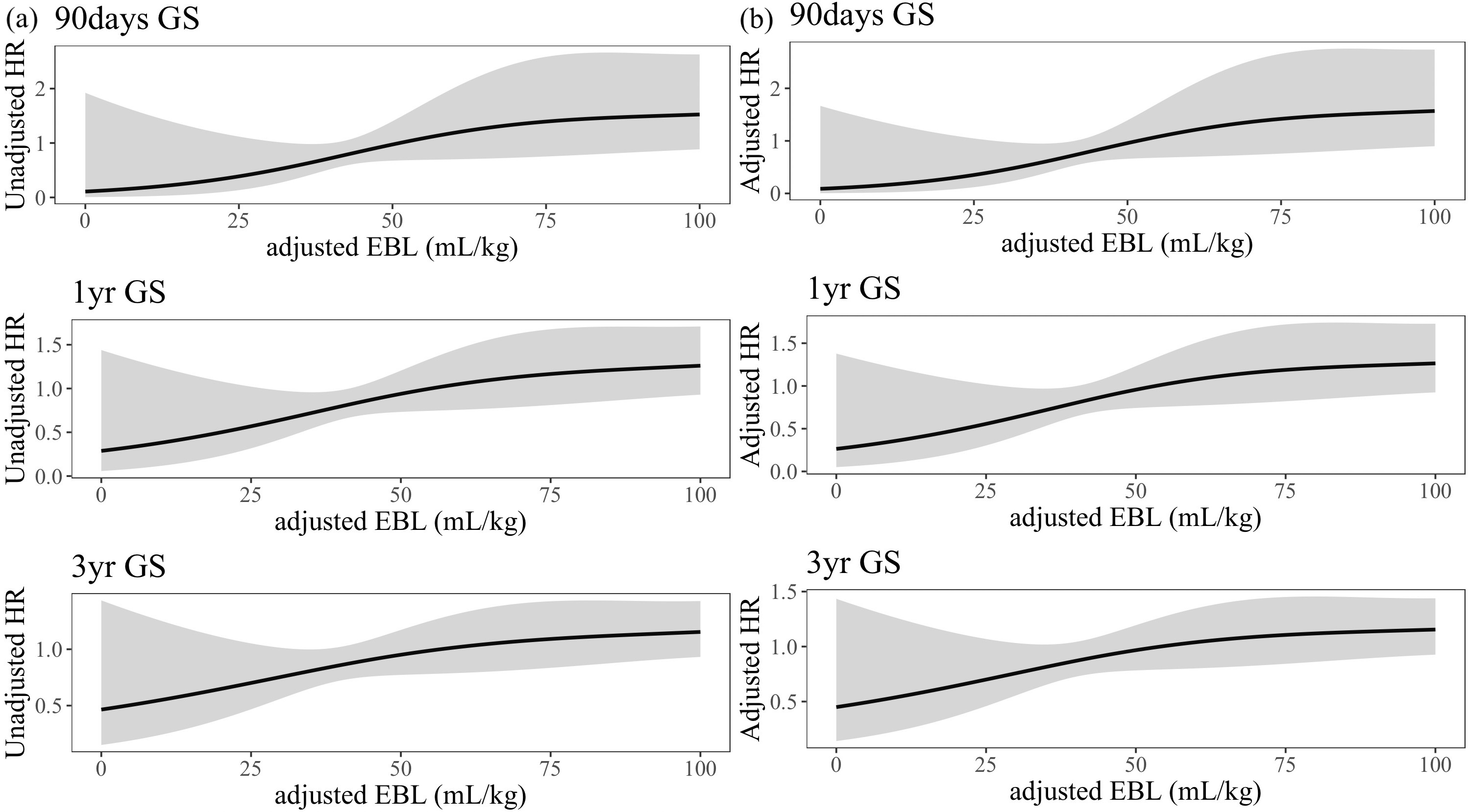

Supplement: Supplementary file 3 — (PNG 749 KB) [file 423_2025_3898_Fig6_ESM.png]

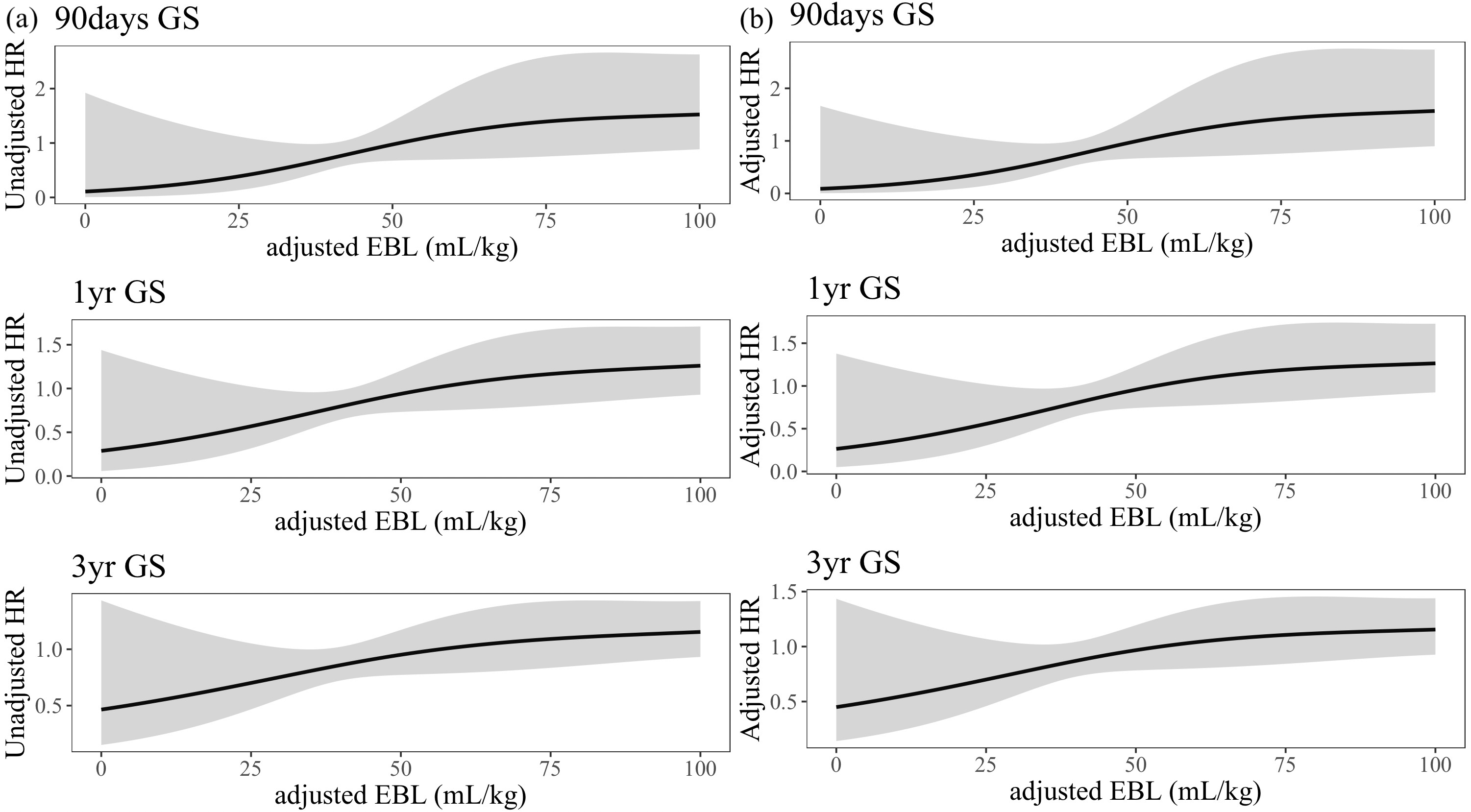

Supplement: Supplementary file 4 — High Resolution Image (TIF 984 KB) [file 423_2025_3898_MOESM2_ESM.tiff]

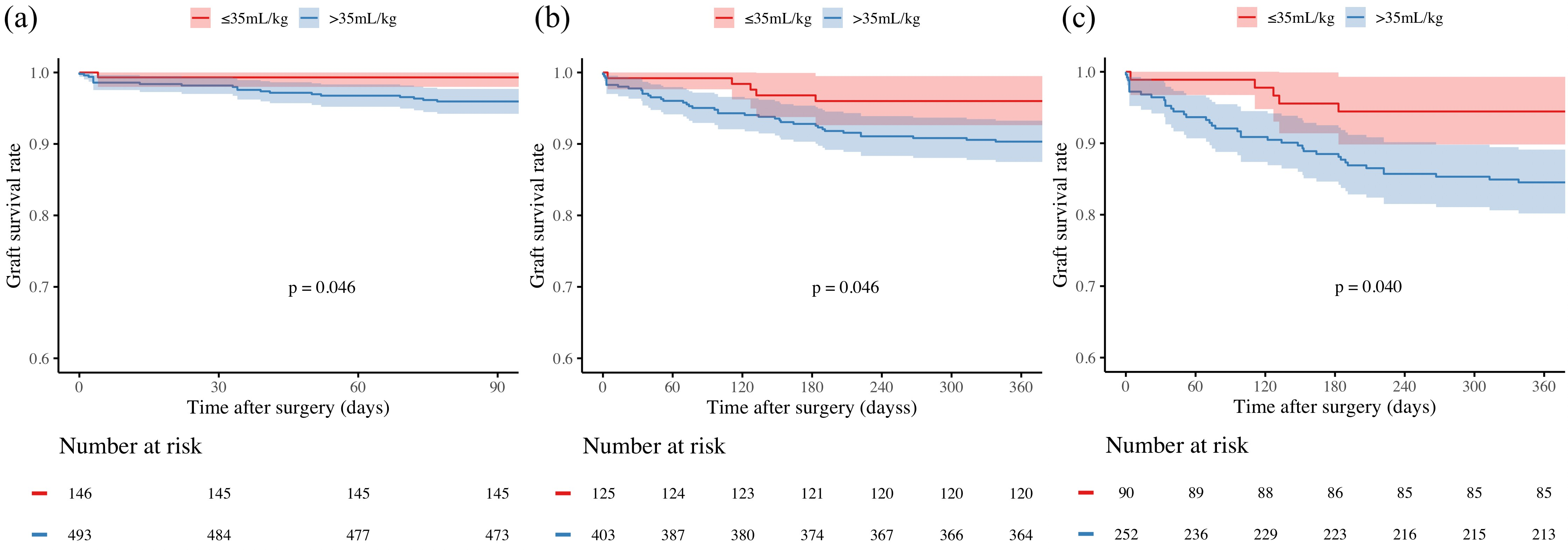

Supplement: Supplementary file 5 — (PNG 737 KB) [file 423_2025_3898_Fig7_ESM.png]

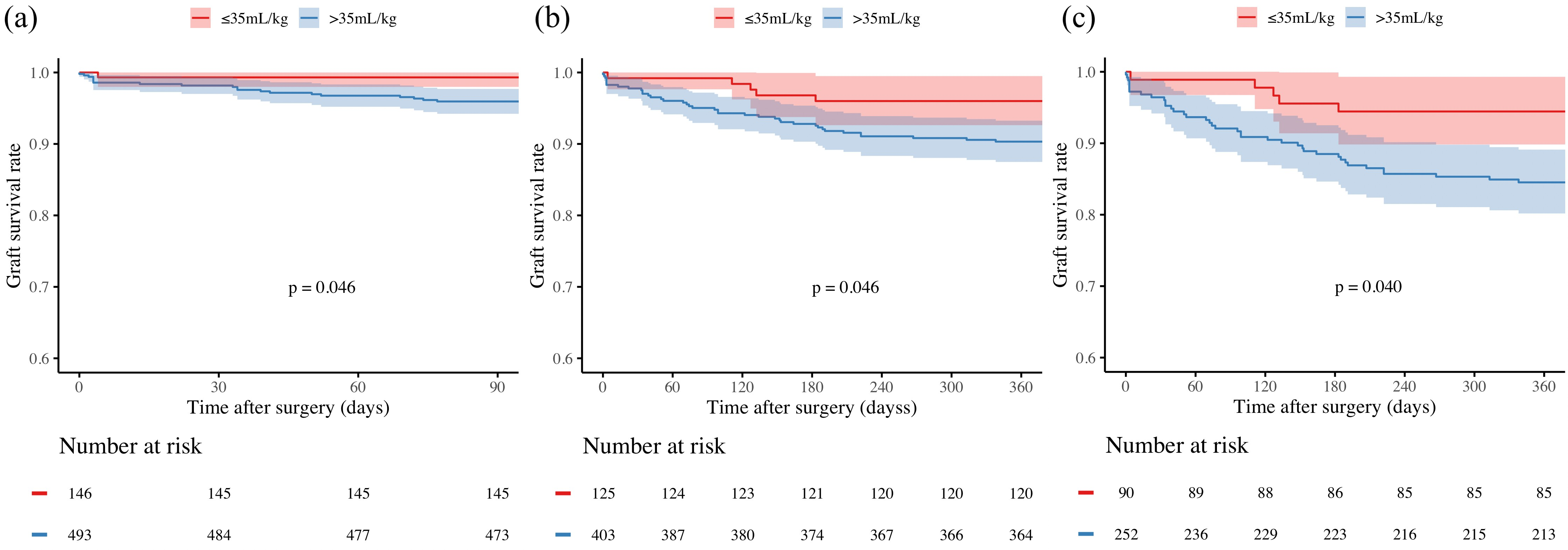

Supplement: Supplementary file 6 — High Resolution Image (TIF 1.28 MB) [file 423_2025_3898_MOESM3_ESM.tiff]

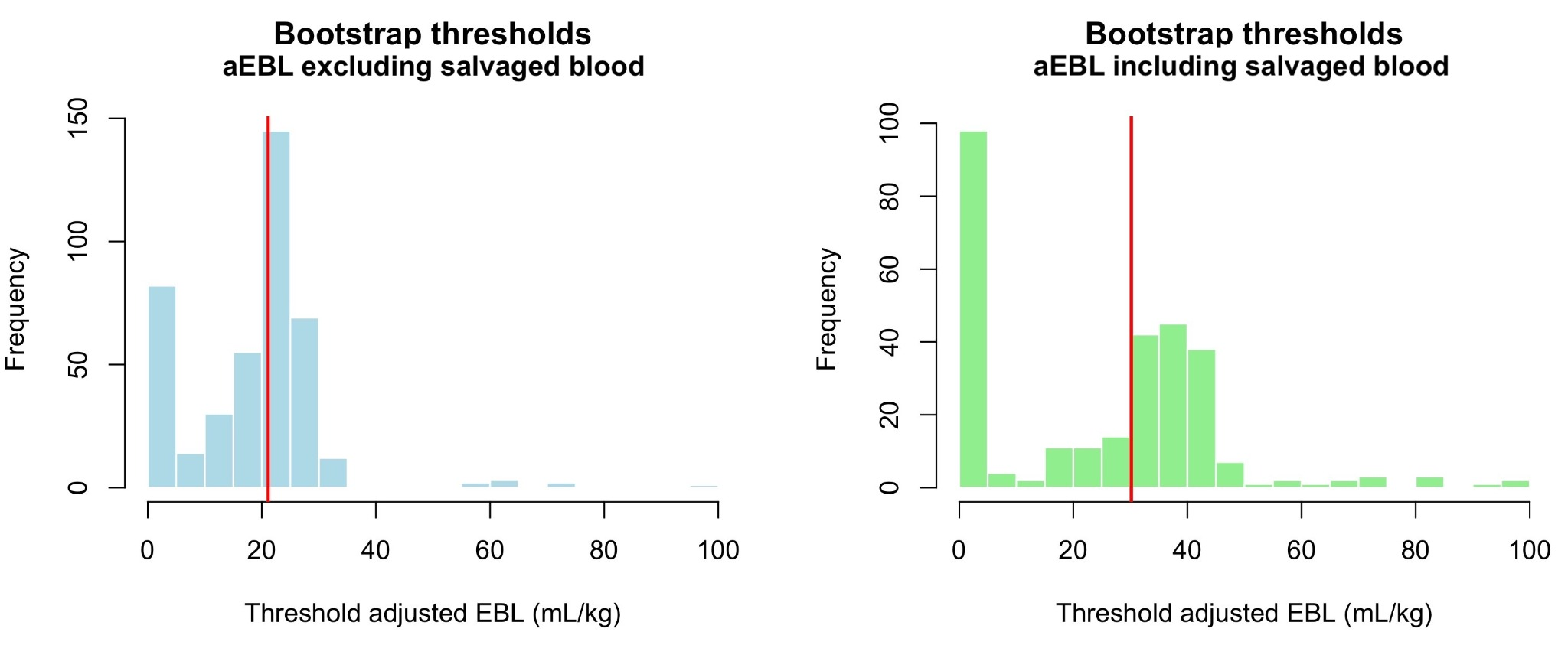

Supplement: Supplementary file 7 — (PNG 248 KB) [file 423_2025_3898_Fig8_ESM.png]

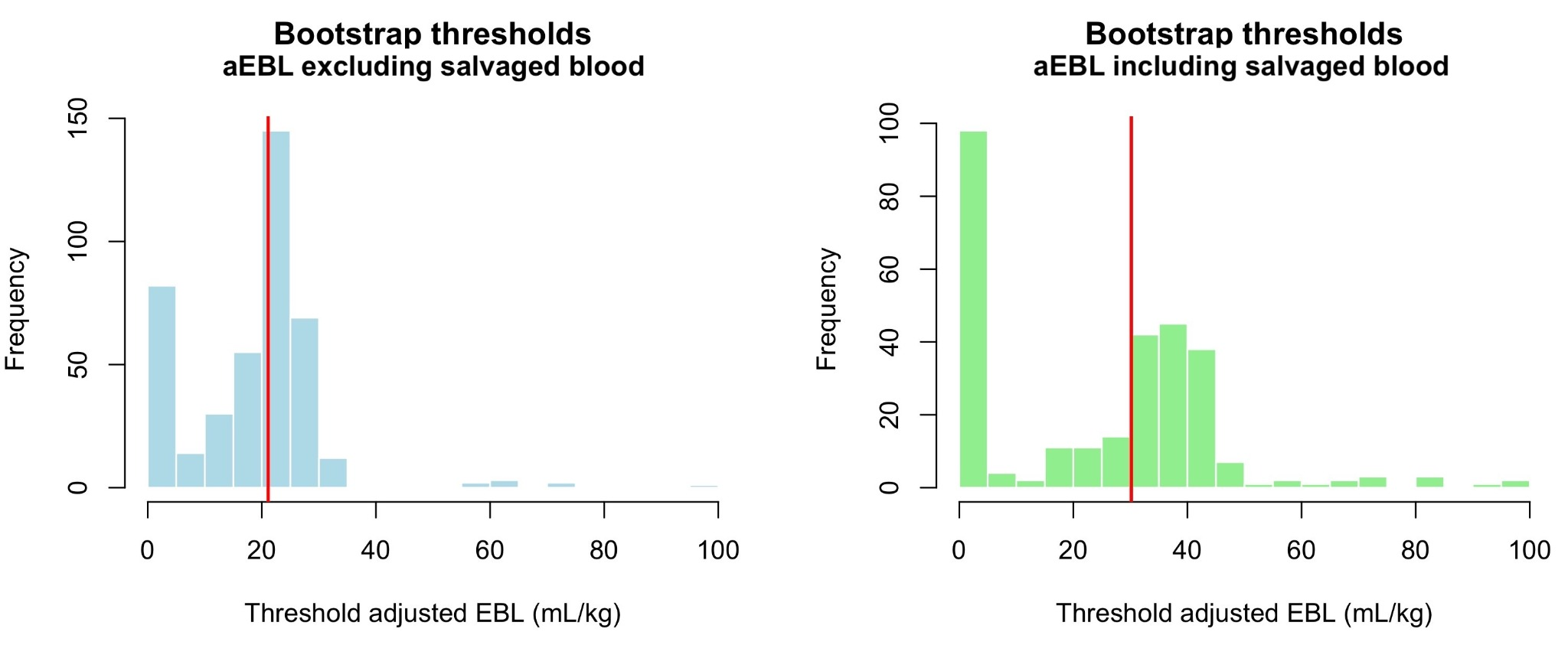

Supplement: Supplementary file 8 — High Resolution Image (TIF 374 KB) [file 423_2025_3898_MOESM4_ESM.tiff]
