# Supplementary material for: Intraoperative blood loss as a predictor of outcomes in liver transplantation: determining optimal cutoff values for improved graft survival
Source: Langenbecks Arch Surg. 2025 Nov 5;411(1):3. doi: 10.1007/s00423-025-03898-z (PMC12589207; doi:10.1007/s00423-025-03898-z)
Supplement: Supplementary file 9 — Supplementary file 5 (20.7 KB) [file 423_2025_3898_MOESM5_ESM.docx]

Supplementary table 1. Intraoperative transfusion requirements by aEBL category (<25.0 vs ≥25.0 mL/kg).

|  | **aEBL < 25.0**  **(N=247)** | **aEBL ≥ 25.0**  **(N=667)** | **Total  (N=914)** | **p-value** |
| --- | --- | --- | --- | --- |
| **Intraoperative transfusion** |  |  |  |  |
| RBC | 6 (3–7) | 7 (5–10) | 7 (4–9) | <0.001 |
| FFP | 2 (2–6) | 6 (4–10) | 6 (4–10) | <0.001 |
| Platelet | 2 (2–2) | 2 (2–4) | 2 (2–4) | <0.001 |
| Cryoprecipitate | 4 (2–4) | 4 (2–5) | 4 (2–4) | 0.02 |

NOTE: Continuous variables: median [IQR]

*Abbreviations.* aEBL: Adjusted estimated blood loss, RBC: Red Blood Cell, FFP: Fresh Frozen Plasma

Supplementary table 2. Multivariate logistic regression analysis to identify risk factors associated with surpassing the aEBL cutoff of 25.0 mL/kg with transfusion

|  | **Odds ratio** | **95% CI** | **p-value** |
| --- | --- | --- | --- |
| **Recipient** |  |  |  |
| Age | 1.00 | 0.99 – 1.02 | 0.77 |
| Male | 0.90 | 0.63 – 1.28 | 0.55 |
| MELD-Na at LT | 1.06 | 1.04 – 1.09 | <0.001 |
| Race |  |  |  |
| Black | Reference |  |  |
| White | 0.53 | 0.22 – 1.26 | 0.15 |
| Hispanic | 0.43 | 0.10 – 1.85 | 0.26 |
| Others | 0.81 | 0.19 – 3.47 | 0.78 |
| PSH of upper abdomen | 1.01 | 0.47 – 2.16 | 0.98 |
| TIPS | 0.61 | 0.32 – 1.14 | 0.12 |
| **Donor** |  |  |  |
| Age | 1.00 | 0.99 – 1.02 | 0.59 |
| BMI, kg/m^2^ | 1.01 | 0.99 – 1.04 | 0.28 |
| CIT | 1.00 | 0.99 – 1.00 | 0.63 |
| HCV antibody positive | 1.68 | 0.98 – 2.90 | 0.06 |
| DCD donor | 2.02 | 1.34 – 3.05 | <0.001 |
| Use of OrganOx | 1.60 | 0.60 – 4.22 | 0.35 |
| RBC (per unit) | 1.11 | 1.06–1.15 | <0.001 |

NOTE: Continuous variables: median [IQR]; Categorical variable: number (%)

*Abbreviations.* aEBL: Adjusted estimated blood loss, BMI: Body mass index, CIT: Cold ischemic time, MELD-Na: Model for End-Stage Liver Disease including Sodium, DCD: Donor from circulatory death, PSH: Past surgical history. TIPS: Transjugular intrahepatic portosystemic shunt, RBC: Red blood cell, HCV: Hepatitis C virus, CI: Confidence interval.
